# Sex differences in the association between sleep duration and muscle quality index in adults: A cross-sectional study from NHANES 2011–2014

**DOI:** 10.1371/journal.pone.0306661

**Published:** 2024-07-15

**Authors:** Bin Xu, MengNi Jiang, Yu Wei, Ran Duan, Feng Tong

**Affiliations:** 1 Urinary Surgery department, Guang Yuan Mental Health Center, Sichuan, China; 2 Clinical Medical College, Chengdu Medical College, Chengdu, Sichuan, China; 3 Department of Cardiovascular, First Affiliated Hospital of Chengdu Medical College, Chengdu, China; 4 Oncology Department, The First Affiliated Hospital of Chengdu Medical College, Chengdu, Sichuan, China; 5 The Second School of Clinical Medicine, Southern Medical University, Guangzhou, China; University of Udine, ITALY

## Abstract

**Objective:**

The aim of this study was to investigate the association between sleep duration and muscle quality index (MQI) in middle-aged and older age groups, as limited evidence exists on this topic.

**Methods:**

In order to assess the relationship between sleep duration and MQI, a cross-sectional study was undertaken, utilizing data from the National Health and Nutrition Examination Survey (NHANES) acquired during the period from 2011 to 2014. The study comprised a total of 4598 participants aged 20 years and above. To examine the association between sleep duration and MQI, sophisticated weighted multivariate linear regression models were employed. Additionally, smooth curve fitting techniques were applied to examine the possibility of any non-linear relationship between the two variables.

**Results:**

The average age of the adults who were enrolled in the study was 38.48±11.69 years, and 46.75% of them were female. The results of the multivariable linear regression models showed that sleep duration had a positive correlation with MQI. However, when subgroup analysis was conducted, it was found that this positive correlation only existed among women (β = 0.09, 95% CI: 0.014 to 0.167). To further confirm the differences between sexes in the relationship between sleep duration and MQI, a weighted generalized additive model (GAM) was used.

**Conclusions:**

This research study provides evidence that there is a positive correlation between the duration of sleep and MQI specifically in females, while no such association was observed in males. These findings shed light on the existence of gender disparities in the connection between sleep duration and MQI.

## Introduction

Sleep is an essential and fundamental aspect of human existence, playing a pivotal role in the overall well-being and optimal functioning of the body. It serves as a cornerstone in maintaining the body’s internal equilibrium, ensuring proper brain function, and regulating the immune system [1]. However, since the 20th century, there has been a significant and consistent decrease in human sleep duration due to lifestyle and work changes [2]. According to the American Academy of Sleep Medicine, it is recommended that adults should aim to get a minimum of 7 hours of sleep each night [3]. Unfortunately, only 48% of American adults manage to sleep between 7 and 9 hours [4].

Skeletal muscle mass and strength are commonly used as indicators of the structural function of skeletal muscle [5]. In the elderly population, there is a significant correlation between decreased skeletal muscle mass and strength, and a higher vulnerability to falls, physical limitations, cognitive decline, metabolic syndrome, cardiovascular disorders, and even mortality [6, 7]. Recently, Lopes, L. et al. introduced the muscle quality index (MQI) as a method to evaluate muscle quality [8]. MQI serves as a predictor of functional capacity and has demonstrated its superiority in assessing muscle function when compared to muscle mass or grip strength alone. It offers a measurable indicator of changes in the locomotive systems and is capable of predicting physical functioning and longevity [9].

There is a strong correlation between a decrease in sleep duration and an increased risk of various diseases. These diseases include cardiovascular disease, chronic kidney disease, neurological abnormalities, type 2 diabetes, cancer, and work-related injuries [7]. Nevertheless, the impact of sleep duration on the MQI in adults remains unclear, and the existing studies have produced inconsistent findings. Certain studies have indicated that poor sleep quality and insufficient sleep duration are linked to decreased muscle mass and strength [10, 11], whereas other studies have reported conflicting results [12, 13]. Further studies have proposed a U-shaped relationship between sleep duration and muscle mass and strength, with an inverted curve [14] or have shown no statistically significant correlation [15]. However, these studies have various limitations, including small sample sizes, insufficient adjustment for critical covariates such as dietary factors [10, 14], physical activity [12], and comorbidity [10, 11], and a narrow focus on older populations [12, 13]. Moreover, the MQI has been recognized as a more accurate indicator of skeletal muscle function compared to muscle mass or hand grip strength [16]. However, until now, no research has explored the direct connection between sleep duration and MQI. Furthermore, it remains unclear whether sex and physical activity may influence this relationship of interest.

In order to fill the existing knowledge gaps, a comprehensive study was carried out to investigate the relationship between sleep duration and MQI among adults aged 20 to 59 years in the United States. Furthermore, an in-depth analysis was conducted to explore any potential variations in these associations between males and females.

## Methods

### Study population

The National Health and Nutrition Examination Survey (NHANES) is a representative survey that has been conducted in the United States to gather comprehensive information about the nutrition and health of the population. The survey uses a dynamic, multi-stage probability sampling design, ensuring that the data collected accurately represents the entire nation.

**Fig 1:**
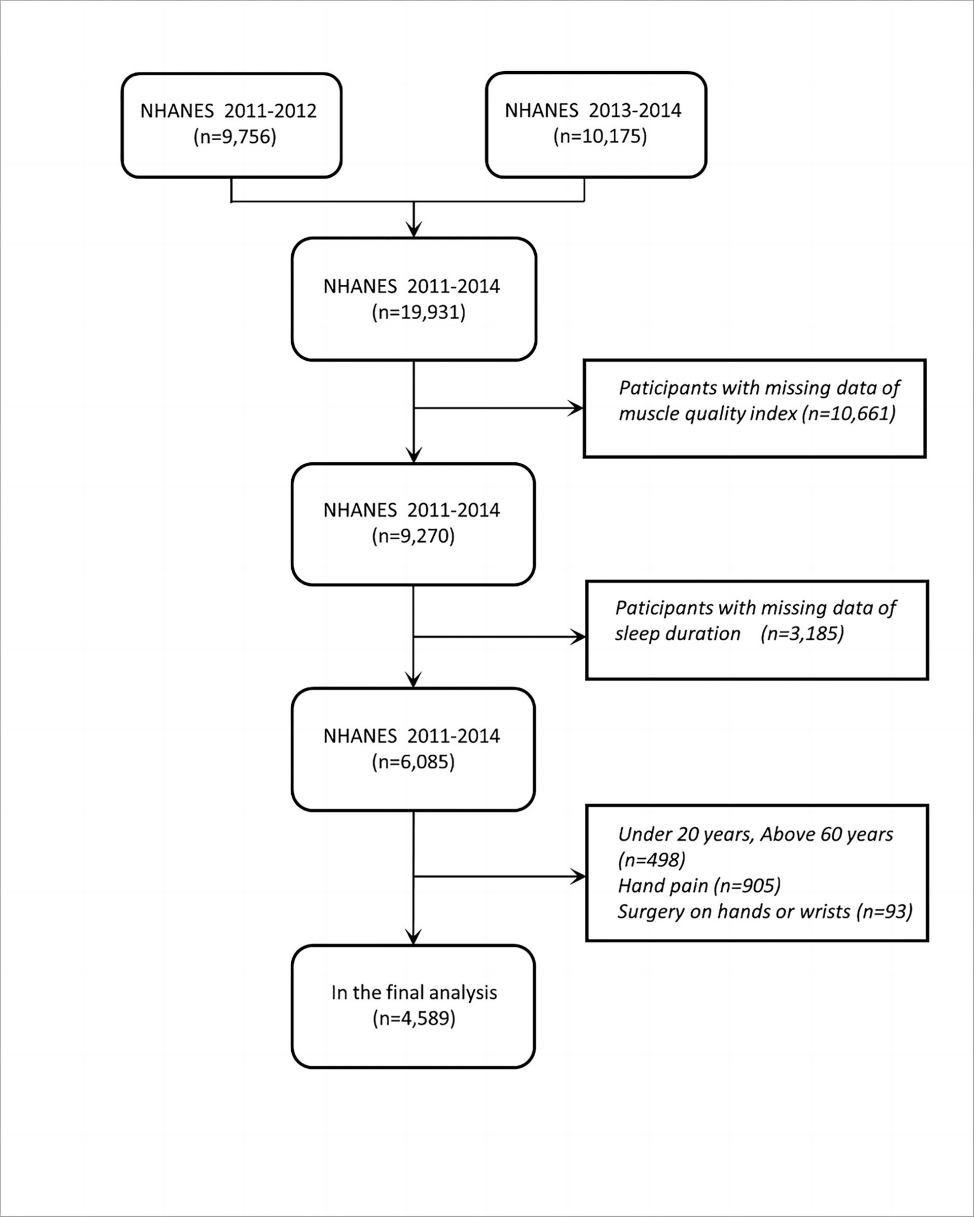
Flow chart of sample selection from the NHANES 2011–2014. This figure illustrates the inclusion and exclusion criteria applied to the initial sample of 19,931 participants, resulting in a final sample of 4,598 participants.

For this particular study, data was extracted and integrated from the NHANES database for the period between 2011 and 2014. The sample size of the study encompassed a total of 19,931 participants. Certain exclusions were applied to the data, specifically participants without MQI data (n = 10,661) or sleep duration data (n = 3,185). Additionally, individuals who were either younger than 20 or older than 60 years (n = 498), those who reported hand pain (n = 905), or those who had undergone wrist surgery (n = 93) were also excluded from the analysis. After accounting for these exclusions, a total of 4,598 participants were included in the final analysis, as presented in Fig 1, which depicts the flow of the study design.

For more detailed information about the survey design, methodology, and data used in this study, interested individuals can visit the NHANES website at https://www.cdc.gov/nchs/nhanes/. It is important to note that the NHANES procedure has received authorization from the National Center for Health Statistics, and all participants involved in the survey have provided written consent.

### Variables

Sleep Assessment: Each participant in the survey was required to fill out a detailed questionnaire evaluating their sleep patterns and wake times during weekdays or workdays. Additionally, the survey included questions about any episodes of intermittent nocturnal awakenings.

In order to measure the skeletal muscle mass of the arm, we utilized dual-energy X-ray absorptiometry (DXA). Furthermore, we employed a Takei dynamometer (TKK 5401; Takei Scientific Instruments, Tokyo, Japan) to measure the strength of the hand grip. Participants were instructed to firmly grip the handle of the meter, and three measurements were taken for both the right and left hands. The value representing the highest grip strength in the dominant hand was utilized as an indicator of the strength of the upper limb skeletal muscles. To determine the magnitude of muscle strength per unit skeletal muscle mass, we defined the ratio of dominant hand grip strength to dominant appendicular skeletal muscle mass as MQI [8].

For the purpose of this study, we carefully selected covariates that are strongly associated with sleep duration and MQI. Detailed information about these covariates was obtained through the administration of a comprehensive questionnaire. The questionnaire encompassed various aspects, such as age (in years), gender, race/ethnicity (including options such as Mexican, American, other Hispanic American, non-Hispanic Black, non-Hispanic White, and other races), education level (differentiating between those with less than a high school education and those with more than a high school education), marital status (including options such as partnered, unpartnered, and unmarried), household poverty-to-income ratio, and a range of health behaviors (such as smoking, alcohol consumption, and physical activity, which was further classified as low, moderate, or vigorous). Additionally, the questionnaire covered various chronic diseases (including depression, arthritis, diabetes, and cardiovascular disease) and dietary intake. When assessing physical activity, the questionnaire considered a wide range of activities, including work-related activities, transportation, leisure activities, and housework. To measure levels of depression, we employed the widely recognized Patient Health Questionnaire-9 (PHQ-9) scale. This scale consists of nine items, each scored on a scale of 0–3. The total score is calculated by summing the scores of the nine items. For the purpose of our study, we categorized the total score as follows: None (0–4), Mild (5–9), Moderate (10–14), Moderately-Severe (15–19), or Severe depression (20–27). This categorization allows us to accurately gauge the severity of depression experienced by participants [17]. The questionnaire calculated the metabolic equivalents (MET) for different physical activities and the total cumulative time spent engaged in each activity per week (in minutes). The individual’s daily level of physical activity was determined by summing these products, measured in MET-min/week [18]. The selection criteria for the covariates were determined based on previously published studies [19, 20]. For the detailed procedure, please refer to: https://wwwn.cdc.gov/nchs/nhanes/Default.aspx.

### Ethical statement

The ethics review board of the National Center for Health Statistics approved all NHANES protocols and written informed consents were obtained from all participants or their proxies (< 18 years).

### Statistical analysis

Multiple group comparisons of all continuous variables in this study were conducted using a weighted linear regression model. The continuous variables were presented as the mean value ± standard deviation. Categorical variables were represented as percentages, and statistical analyses were performed using weighted chi-square tests to compare multiple groups. To explore the association between sleep duration and MQI, a weighted multivariate linear regression analysis was employed. In Model 1, no covariates were adjusted. Model 2 included adjustments for potential confounders such as age, sex, and race. Model 3 accounted for multiple potential confounders, including education level, marital status, income to poverty ratio, smoking status, alcohol use, depression level, MET scores, diabetes, arthritis, cardiovascular disease, energy intake (kcal/day), protein intake (g/day), carbohydrate intake (g/day), total fat intake (g/day), and caffeine intake (mg/day). Subgroup analyses were conducted based on Model 3 to examine whether the relationship between sleep duration and MQI was influenced by gender or physical activity. Moreover, the potential non-linear relationships between sleep duration and MQI were assessed using a smoothing curve model. The significance level for all analyses was set at 0.05.

## Results

### Study sample

Table 1 presents detailed information regarding the weighted demographic characteristics, health conditions, behavior, and dietary intake in this study. The total number of individuals included in the study was 4,589, with an average age of 38.5±11.7 years. Among the participants, 46.8% were female. It is worth noting that 39.81% of the study subjects reported sleeping for six hours or less, while 5.62% reported sleeping for nine hours or more.

**Table 1:** Characteristic of study sample.

Characteristic	Total (n = 4598)	≤6h (n = 1833)	7-8h (n = 2378)	≥9h (n = 387)	*P* value between-groups
Demographic					
Age (years)	38.48±11.69	38.59±11.81	38.52±11.81	35.46±11.71	<**0.001**
Sex (%)					**0.004**
Male	53.25	55.28	52.76	44.51	
Female	46.75	44.72	47.24	55.49	
Race (%)					<**0.001**
Non-Hispanic White	62.86	59.26	65.35	61.68	
Non-Hispanic Black	11.61	15.63	9.24	8.62	
Mexican American	10.37	10.12	10.06	15.22	
Other Hispanic	15.16	14.98	15.35	14.49	
Marital status(%)					<**0.001**
Never married	26.49	26.32	25.96	32.97	
Married	51.44	48.51	54.42	40.83	
Widowed	1.07	1.46	0.91	10.82	
Divorced	9.18	10.42	8.2	13.28	
Living with partner	9.28	9.65	8.65	2.1	
Separated	2.53	3.63	1.85	-	
Education (%)					<**0.001**
Less than high school	13.42	14.65	12.42	15.3	
High school	20.51	23.04	18.82	20.65	
More than high school	66.08	62.31	68.76	64.05	
Income to poverty ratio (%)					<**0.001**
<1	47.47	43.37	51.43	34.72	
1–3	31.23	34.33	29.1	32.06	
>3	16.1	17.43	14.36	24.92	
Not record	5.19	4.87	5.1	8.29	
**Health Conditions and Behavior**					
Smoking behavior (%)					<**0.001**
None	61.03	56.47	64.8	53.32	
Past	17.61	16.95	18.22	15.86	
Current	21.33	26.51	16.98	30.82	
Not record	0.02	0.06	-	-	
Alcohol consumption (%)					**0.004**
None	9.71	8.99	10.09	10.83	
Past	9.2	10.78	7.9	11.86	
Mild	31.71	31.05	32.54	27.73	
Moderate	18.99	17.3	20.53	14.63	
Heavy	26.15	27.83	24.65	30.16	
Not record	4.23	4.05	4.3	4.79	
MET scores (%)					<**0.001**
Mild	34.17	39.82	31.04	27.99	
Moderate	36.28	30.42	40.25	35.23	
Heavy	13.53	12.17	14.37	14.2	
Not record	16.02	17.6	14.34	22.58	
Diabetes(%)					0.067
No	94.53	93.53	95.17	94.81	
Yes	5.47	6.47	4.83	5.19	
Cardiovascular disease(%)					**0.021**
No	97.22	96.33	97.81	98.17	
Yes	2.78	3.67	2.19	1.83	
Arthritis					**0.004**
No	90.34	88.33	91.81	88.95	
Yes	9.65	11.64	8.19	11.05	
Not record	0.01	0.03	-	-	
Depression level					<**0.001**
None	77.2	71.52	81.61	70.45	
Mild	12.39	16.49	9.41	15.09	
Moderate	3.86	4.92	3.14	4.05	
Moderately-Severe	1.57	2.09	0.93	4.6	
Severe	0.62	0.87	0.42	0.97	
Not record	4.37	4.12	4.49	4.84	
**Dietary Intake**					
Energy (kcal/day)	2220.78±862.53	2239.28±921.34	2214.16±818.10	2163.58±890.48	0.355
Protein (g/day)	86.84±37.45	86.96±39.30	87.15±36.14	82.84±37.52	0.211
Carbohydrate (g/day)	265.99±110.62	269.85±118.06	264.01±104.75	260.17±116.17	0.161
Total fat (g/day)	84.21±39.72	85.43±42.05	83.83±38.44	79.87±35.80	0.0851
Caffeine (mg/day)	156.70±171.55	164.27±179.81	154.87±169.40	124.26±125.83	**0.002**

Mean ± SD for continuous variables: P value was calculated by weighted linear regression model

Percent for categorical variables: P value was calculated by weighted chi-square test

The results of our analysis revealed statistically significant differences (P<0.001) in various factors across different sleep duration groups. The factors that showed significant differences included age, sex, race, education level, marital status, income to poverty ratio, smoking status, alcohol use, MET scores, depression level, diabetes, arthritis, cardiovascular disease, energy intake, protein intake, carbohydrate, total fat, and caffeine.

### Association between sleep duration and MQI

**Fig 2:**
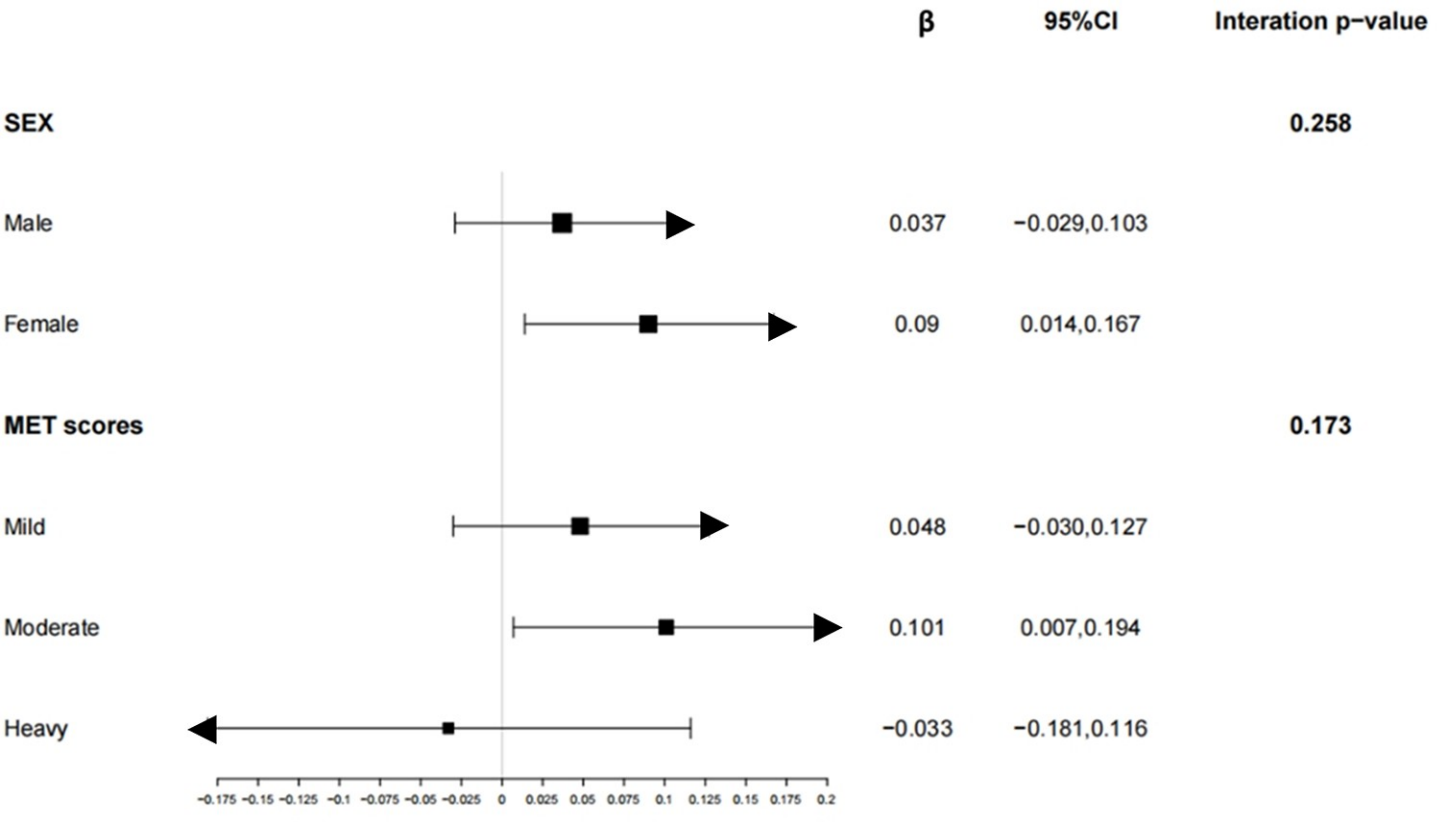
Associations between sleep duration and MQI stratified by sex and MET scores. The figure shows the positive correlation between sleep duration and MQI, with separate lines for males and females, as well as for different levels of physical activity (low, moderate, high). Adjusted for age, sex, race, education level, marital status, income to poverty ratio, smoking status, alcohol use, depression level, MET scores, depression level, diabetes, arthritis, cardiovascular disease, energy intake (kcal/day), protein intake (g/day), carbohydratel (g/day), total fat (g/day) and caffeine (mg/day).

**Fig 3:**
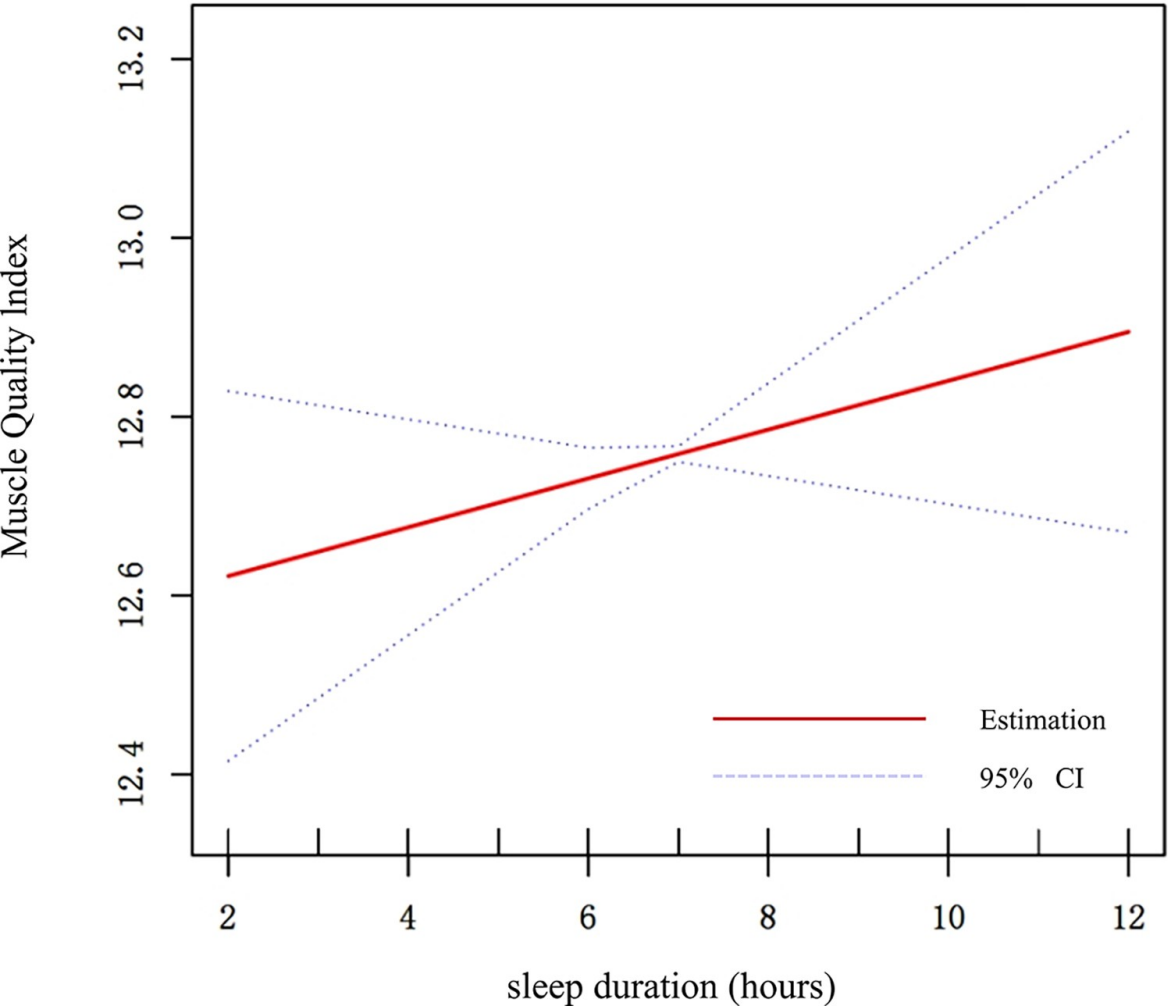
The association between sleep duration and MQI. The X-axis represents sleep duration (hours), and the Y-axis represents the Muscle Quality Index (MQI). The blue bands represent the 95% confidence interval for the fitted line, adjusted for age, sex, race, education level, marital status, income to poverty ratio, smoking status, alcohol use, depression level, MET scores, diabetes, arthritis, cardiovascular disease, energy intake (kcal/day), protein intake (g/day), carbohydrate intake (g/day), total fat intake (g/day), and caffeine intake (mg/day).

**Fig 4:**
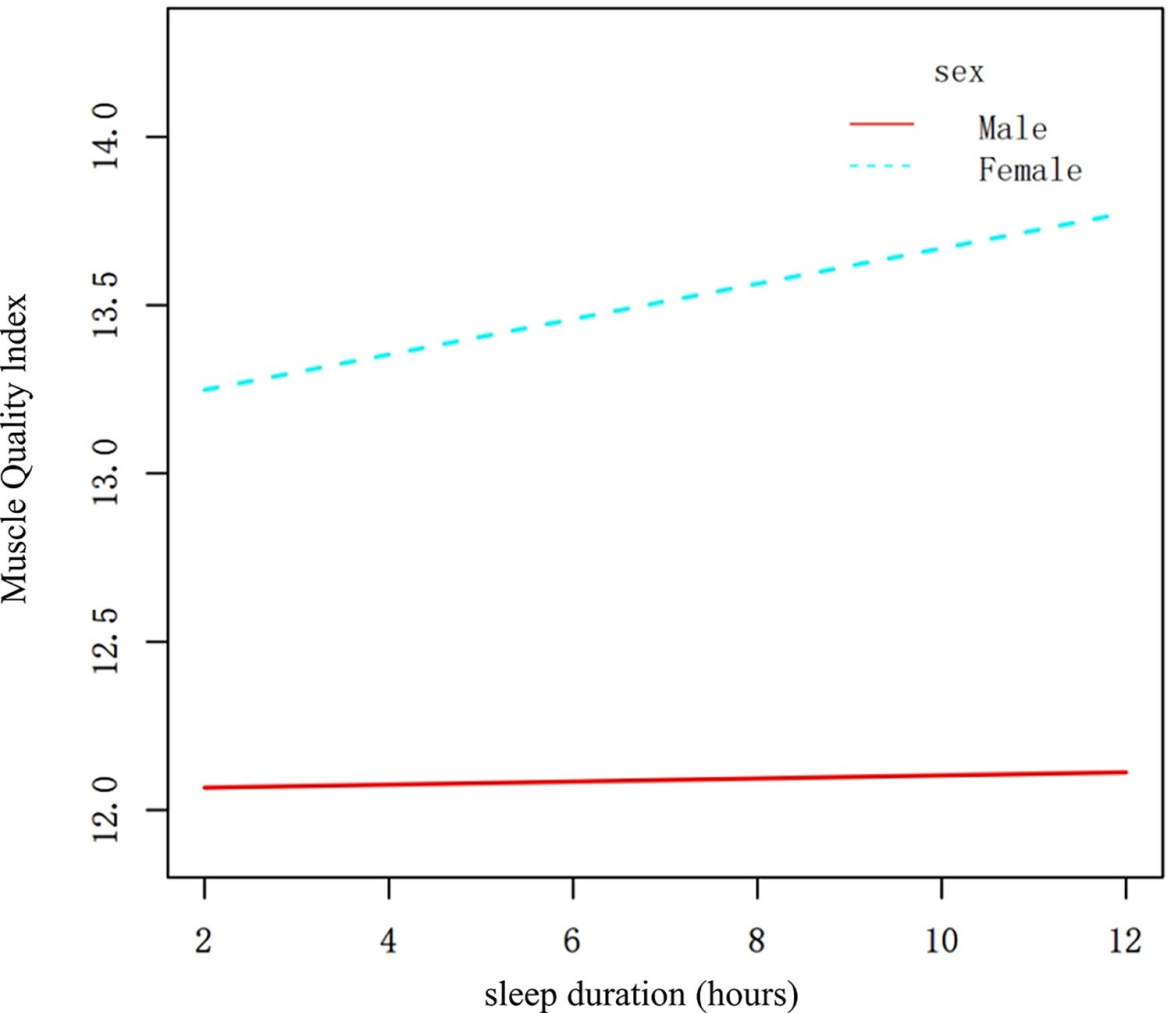
The association between sleep duration and MQI stratified by sex. The X-axis represents sleep duration (hours), and the Y-axis represents the Muscle Quality Index (MQI). This figure shows the different trends in the association between sleep duration and MQI for males and females, adjusted for the same covariates as Fig 3.

Sleep duration exhibited a positive association with MQI in the multivariable linear regression models (Table 2). Furthermore, in the subgroup analyses, this positive association was observed specifically in women (β = 0.09, 95% CI: 0.014 to 0.167) and individuals with moderate MET scores (β = 0.101, 95% CI: 0.007 to 0.194) (Fig 2). To validate these results, a weighted generalized additive model (GAM) was employed, which confirmed the relationship between sleep duration and MQI (Figs 3 and 4).

**Table 2:** Associations between sleep duration and MQI.

	N	Model 1 β (95% CI)	Model 2 β (95% CI)	Model 3 β (95% CI)
sleep duration (h)	4598	0.136(0.082,0.190)	0.081(0.031,0.131)	0.064(0.014,0.115)
Duration of sleep (h)				
≤6h	1833	Reference	Reference	Reference
7-8h	2378	0.319(0.174,0.464)	0.224(0.089,0.359)	0.133 (-0.002,0.268)
≥9h	387	0.563 (0.251,0.875)	0.284(-0.007,0.575)	0.334(0.046,0.621)
P for trend		<0.001	<0.001	0.012

Model 1: no covariates were adjusted

Model 2: age, sex, and race were adjusted

Model 3: age, sex, race, education level, marital status, income to poverty ratio, smoking status, alcohol use, depression level, MET scores, depression level, diabetes, arthritis, cardiovascular disease, energy intake (kcal/day), protein intake (g/day), carbohydratel (g/day), total fat (g/day) and caffeine (mg/day) were adjusted

## Discussion

This study used a cohort of American subjects to examine the relationship between sleep duration and MQI. Our study suggested elevated sleep duration correlated with higher MQI in women.

Numerous epidemiological studies have sought to establish a correlation between sleep duration and MQI. However, these studies have yielded conflicting findings, leading to inconsistent conclusions. In a study of 10,125 university students from China, Chen et al. found a significant association between lower skeletal muscle strength, estimated by a handheld digital dynamometer, and decreased sleep duration [10]. However, it is important to acknowledge that this adjustment was not exhaustive, as their analysis did not consider other significant factors like dietary choices, level of physical activity, and presence of other medical conditions. Consequently, while their initial findings imply a potential link between sleep duration and MQI, the accuracy and reliability of these conclusions may be questioned due to the closely intertwined nature of these confounding factors with MQI. Likewise, the findings from the Berlin Aging Study II indicated a positive correlation between sleep quality and both skeletal muscle mass and strength in women [21]. While this particular study had a distinct population and methodology, it displayed similar limitations. Moreover, a majority of prior investigations focused on older individuals. However, there is scarce evidence concerning the relationship between sleep duration and MQI in middle-aged and older age groups. Our study, which encompassed 4598 adults in the United States, sought to examine the connections between sleep duration and MQI. We considered various factors in our analysis, including sex, race/ethnicity, education level, marital status, physical activity, comorbidities, and dietary intake. The inclusion of a large number of participants enhanced the statistical power of our study, allowing for reliable and consistent findings. Additionally, the data collection process, which relied on NHANES data, ensured a consistent and standardized approach in gathering clinical and dietary intake information.

Sleep plays a crucial role in regulating various hormones that influence muscle metabolism. Adequate sleep is associated with optimal levels of growth hormone and insulin-like growth factor 1 (IGF-1), both of which are critical for muscle growth and repair [22, 23]. Hypercortisolism promotes protein degradation, inhibits protein synthesis, impairs muscle structure, and ultimately causes muscle atrophy [24]. IGF-1 promotes myogenic cell differentiation and muscle production, thereby maintaining muscle mass [25]. IGF-1 promotes the differentiation of myogenic cells and the production of muscles, thus preserving muscle mass. Insulin enhances muscle anabolism and inhibits muscle degradation, whereas shorter sleep duration induces insulin resistance through various metabolic pathways [26]. Chronic sleep deprivation can elevate pro-inflammatory cytokines such as interleukin-6 (IL-6) and tumor necrosis factor-alpha (TNF-α). These inflammatory markers have been shown to negatively impact muscle health by promoting muscle catabolism and reducing muscle anabolism [27]. Insufficient sleep is linked to insulin resistance, which can impair muscle glucose uptake and glycogen synthesis, essential processes for muscle energy and function. Insulin resistance can also lead to an imbalance in protein metabolism, favoring muscle protein breakdown over synthesis [26].

Gender is a significant factor that plays a role in influencing both muscle quality and sleep health independently. Analyzing the divergences in muscle quality and sleep issues between males and females can provide valuable information for understanding healthcare utilization, prevention strategies, and treatment approaches for these disorders. Our findings suggest that adhering to a recommended sleep duration is advantageous for females as it reduces the likelihood of developing sarcopenia. It is crucial not to disregard the disparities in sleep problems according to gender, as it is commonly observed that women face higher rates of difficulty in achieving sound sleep [28, 29]. Evidence suggests that hormonal steroids and changes during menopause play significant roles in this condition [30]. Several previous reports have shown that grip strength decreases during both the follicular and luteal phases [31]. Furthermore, delayed muscle recovery was observed in females during the menstrual phase [32]. It is worth considering that menstrual cycles and female hormones may be significant mediators that influence the relationship between sleep duration and MQI in females.

This research study possesses several notable advantages. Firstly, the utilization of the NHANES public database as the source for our study population ensures a significantly large sample size, as well as a consistent collection of pertinent indicators and variables. This aspect greatly enhances the robustness and reliability of our findings. Secondly, we have employed two distinct statistical models, namely the generalized linear model and the generalized additive model, to thoroughly evaluate both linear and nonlinear relationships between sleep duration and MQI (muscle quality index). By employing these models, we are able to provide a more accurate depiction of the association between sleep duration and MQI, thereby enhancing the credibility and validity of our results. Thirdly, it is imperative to acknowledge that this study falls under the category of purely observational research, which inherently introduces the possibility of potential confounding factors. However, we have taken comprehensive measures to systematically adjust for such factors, thereby strengthening the research methodology and improving upon the limitations of previous studies conducted in this domain. Additionally, the inclusion of subgroup analysis and interaction testing further augments the precision and accuracy of our findings. Specifically, through subgroup analysis, we have unveiled a positive association between sleep duration and MQI in females, while this relationship is not observed in males. This important discovery allows for a nuanced understanding of the gender-specific impact of sleep duration on muscle quality. Furthermore, the inclusion of the MQI, as opposed to solely relying on muscle mass or grip strength, has proven to be a more robust indicator of muscle quality.

The findings of this study hold value when it comes to concerns related to sleep health. It has provided valuable insights for healthcare professionals, revealing a close association between sleep duration and muscle quality. Consequently, it has paved the way for early detection and proper management of sleep health issues in clinical settings. In order to prevent or improve trouble sleeping, assessments and interventions should now consider muscle quality as a new and strong predictor. Nowadays, non-pharmacological methods of managing trouble sleeping are becoming increasingly popular, with regular physical or resistance exercises being recognized as effective countermeasures to address sleep problems.

There are also some limitations. First, as a cross-sectional study, it can only provide clues for causal associations, but not causal inferences. Second, the sleep duration in this study were all obtained through questionnaires based on the recall of the respondents, which may be subject to recall bias. Third, computed tomography and magnetic resonance imaging are the most accurate methods for measuring muscle mass, but they are costly, time-consuming, and cannot be analyzed in bulk, making them difficult to apply in large-scale epidemiological studies. DXA is less accurate than computed tomography and magnetic resonance imaging, but DXA has the advantages of relatively lower cost and shorter scan time, making it the most widely used method. Finally, the NHANES 2011–2014 survey included only average hours of sleep duration. It would be interesting to combine nap duration and nighttime sleep duration to assess their effect on MQI.

## Conclusions

Sleep is a significant issue pertaining to public health in the United States. The results demonstrated that there was no noteworthy correlation between sleep duration and MQI among males. However, among females, a positive link was identified between sleep duration and MQI. To gain a better understanding of this inequality, it is recommended that future longitudinal investigations delve into the factors that contribute to it and explore the underlying mechanisms. This knowledge could ultimately aid in the development of interventions aimed at enhancing MQI.

## Abbreviations

MQI: muscle quality index
DXA: Dual-energy X-ray absorptiometry
NHANES: National Health and Nutrition Examination Survey
GAM: generalized additive model
MET: metabolic equivalent task
IGF-1: insulin-like growth factor
